# Consistent Metagenome-Derived Metrics Verify and Delineate Bacterial Species Boundaries

**DOI:** 10.1128/mSystems.00731-19

**Published:** 2020-01-14

**Authors:** Matthew R. Olm, Alexander Crits-Christoph, Spencer Diamond, Adi Lavy, Paula B. Matheus Carnevali, Jillian F. Banfield

**Affiliations:** a Department of Earth and Planetary Science, University of California, Berkeley, Berkeley, California, USA; b Department of Plant and Microbial Biology, University of California, Berkeley, Berkeley, California, USA; c Department of Environmental Science, Policy, and Management, University of California, Berkeley, Berkeley, California, USA; d Earth Sciences Division, Lawrence Berkeley National Laboratory, Berkeley, California, USA; e Chan Zuckerberg Biohub, San Francisco, California, USA; DOE Joint Genome Institute

**Keywords:** bacterial species, bioinformatics, metagenomics, microbial genetics, species

## Abstract

Longstanding questions relate to the existence of naturally distinct bacterial species and genetic approaches to distinguish them. Bacterial genomes in public databases form distinct groups, but these databases are subject to isolation and deposition biases. To avoid these biases, we compared 5,203 bacterial genomes from 1,457 environmental metagenomic samples to test for distinct clouds of diversity and evaluated metrics that could be used to define the species boundary. Bacterial genomes from the human gut, soil, and the ocean all exhibited gaps in whole-genome average nucleotide identities (ANI) near the previously suggested species threshold of 95% ANI. While genome-wide ratios of nonsynonymous and synonymous nucleotide differences (*dN*/*dS*) decrease until ANI values approach ∼98%, two methods for estimating homologous recombination approached zero at ∼95% ANI, supporting breakdown of recombination due to sequence divergence as a species-forming force. We evaluated 107 genome-based metrics for their ability to distinguish species when full genomes are not recovered. Full-length 16S rRNA genes were least useful, in part because they were underrecovered from metagenomes. However, many ribosomal proteins displayed both high metagenomic recoverability and species discrimination power. Taken together, our results verify the existence of sequence-discrete microbial species in metagenome-derived genomes and highlight the usefulness of ribosomal genes for gene-level species discrimination.

**IMPORTANCE** There is controversy about whether bacterial diversity is clustered into distinct species groups or exists as a continuum. To address this issue, we analyzed bacterial genome databases and reports from several previous large-scale environment studies and identified clear discrete groups of species-level bacterial diversity in all cases. Genetic analysis further revealed that quasi-sexual reproduction via horizontal gene transfer is likely a key evolutionary force that maintains bacterial species integrity. We next benchmarked over 100 metrics to distinguish these bacterial species from each other and identified several genes encoding ribosomal proteins with high species discrimination power. Overall, the results from this study provide best practices for bacterial species delineation based on genome content and insight into the nature of bacterial species population genetics.

## Abstract (precis)

There is controversy about whether bacterial diversity is clustered into distinct species groups or exists as a continuum. To address this issue, we analyzed bacterial genome databases and reports from several previous large-scale environment studies and identified clear discrete groups of species-level bacterial diversity in all cases. Genetic analysis further revealed that quasi-sexual reproduction via horizontal gene transfer is likely a key evolutionary force that maintains bacterial species integrity. We next benchmarked over 100 metrics to distinguish these bacterial species from each other and identified several genes encoding ribosomal proteins with high species discrimination power. Overall, the results from this study provide best practices for bacterial species delineation based on genome content and insight into the nature of bacterial species population genetics.

## INTRODUCTION

A fundamental issue of microbiology is whether bacterial genetic diversity exists as a continuum or is divided into distinct clusters (1–4). A number of previous studies have shown that environmental DNA fragments are either closely related to or unrelated to other sequences from the same environment (4–7), providing evidence for the existence of sequence-discrete populations. However, whether genomes from environmental samples (genome sets unbiased by targeted analyses) tend to cluster into distinct groups has not yet been analyzed on a genome-wide basis and at scale. Discrete sequence populations have been identified in large public genome databases (8, 9), most recently in a study using ∼90,000 bacterial genomes available in the public NCBI Genome database as of March 2017 (10). In those studies, genomes most commonly shared either >97% or <90% average nucleotide identity (ANI). A bacterial species threshold of 95% ANI, originally proposed on the basis of benchmarking with respect to DNA-DNA hybridization values (8), has been gaining increasing support (11) on the basis of that observation. However, it is still unclear whether this pattern is confounded by database biases or whether it reflects a true phenomenon across natural environments, as comparisons of phylogenetically unbalanced genome sets could result in the formation of spurious sequence clusters.

Over 75% of the genomes with assigned taxonomy in the NCBI Genome database are from the *Proteobacteria* and *Firmicutes* phyla, and over 10% are from the genus *Streptococcus* alone (10). Attempts have been made to remove the bias from this reference genome set in searching for naturally distinct bacterial populations, for example, by sampling five genomes from each species with at least five genomes in the database (10), but selective cultivation and sequencing cause biases that are difficult to account for. Biases introduced in the databases include those resulting from sequencing and depositing isolates that meet the expected criteria of target species and those resulting from cultivation with selective media that favor certain genotypes and suppresses the growth of alternative ones. Sets of genomes without selection and cultivation biases can be acquired through the direct sequencing of environmental DNA (genome-resolved metagenomics). While metagenomic sequencing suffers from its own biases, including better DNA extraction from Gram-negative than from Gram-positive bacteria (12, 13), it is unlikely that this kind of broad bias would contribute to patterns of species-level sequence groups. The set of genomes that can be assembled from metagenomes can also be biased. For example, genome recovery may be precluded when multiple similar genomes are present in the same sample (14, 15). However, these strain-level biases should not affect the ability to resolve species-level groups.

If distinct microbial species exist, a relatively comprehensive analysis of public data may uncover the roles that recombination and selection play in their origin. Several hypotheses have been proposed to explain genetic discontinuities, including a decrease in the rates of homologous recombination at the species threshold (16, 17), periodic selective events that purge genetic diversity (18), and neutral processes (19). Computer simulations suggest that both homologous recombination and selection are needed to form genotypic clusters (20), and quantitative population genomic analyses of metagenomics data point to the declining rates of homologous recombination concurrent with sequence divergence as the force behind the clustering (21). While compelling descriptions of speciation have been shown for a limited number of organisms (22, 23), homologous recombination and selective pressures have not been measured and analyzed at scale across thousands of genomes or in direct relation to the proposed 95% ANI species threshold. A common method of detecting selection is use of the *dN*/*dS* ratio, or the ratio of nonsynonymous (*dN*) to synonymous (*dS*) nucleotide changes. Deviations from an expected 1:1 ratio can indicate selective pressures, as nonsynonymous mutations usually have a greater impact on phenotype and are thus more likely to represent targets of selection than synonymous mutations. Whole-genome comparisons of *dN*/*dS* data nearly always result in values below 1 (24), indicative of purifying selection and likely due to the continuous removal of slightly deleterious nonsynonymous mutations over time.

To understand the species composition of a microbial environment, it is essential to be able to accurately assign sequences to species clusters. While metagenome-assembled genomes (MAGs) can be compared using whole-genome ANI, only a fraction of assembled scaffolds are binned from complex environmental metagenomes. For example, only 24.2% of the reads could be assembled and binned in a recent study of permafrost metagenomes (25). More recently, only 36.4% of reads were assembled into binned contigs, and genomes were reconstructed for only ∼23% of the detected bacteria in complex soil metagenomes (26). Absent dramatic improvements in sequencing technologies, complex communities can be more fully characterized through analysis of assembled and unbinned single-copy marker genes, for example, the 16S rRNA gene, ribosomal genes, or tRNA-ligase genes (27–30). It is known that less-conserved marker genes display consistent phylogenetic signals (31) and can outperform the 16S rRNA gene for species delineation of specific taxa (32), but the accuracy and identity thresholds of these genes for generalized species delineation, as well as their ability to be assembled from metagenomic data, are unknown. It is therefore important to identify marker genes that not only accurately reflect change in species taxonomy and divergence but also assemble often in metagenomes using common next-generation sequencing technologies.

Here, we analyzed thousands of bacterial genomes recovered directly from the sequencing of environmental DNA to test for the existence of discrete sequence clusters, developed software to estimate the strength of recombination and selection forces operating between these genomes, and compared over a hundred marker genes for practical species delineation. Discrete sequence clusters were identified in all environments tested, and both estimated recombination rates and genome-wide *dN*/*dS* ratios showed clear patterns in relation to the 95% ANI species threshold. Whole-genome ANI methods were compared to various marker gene alignments (including 16S rRNA) for the ability to create species-level groups, and optimal species delineation thresholds were calculated for each method. Overall, our results support the idea of the existence of discrete species-level groups for bacteria in the three divergent environments tested, provide sequence-based evidence for the likely evolutionary forces at play, and provide metrics for species delineation in metagenomics studies.

(Part of the manuscript was previously reported in the thesis of author M. R. Olm.)

## RESULTS

### Discrete sequence groups exist in all analyzed genome sets.

**FIG 1:**
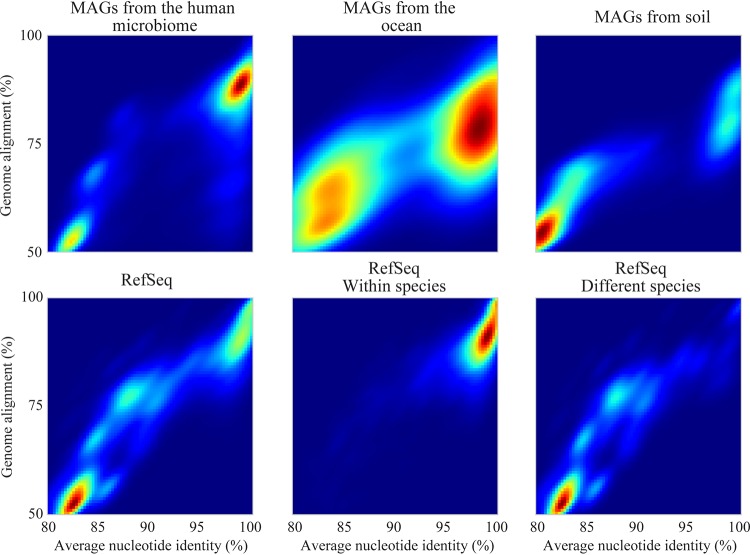
Average nucleotide identity gaps exist near ∼95% ANI in all tested genome sets. Each plot is a histogram of average nucleotide identity and genome alignment percentage values resulting from pairwise comparison within a genome set. Higher-intensity colors represent a higher density of comparisons with that particular ANI and genome alignment percentage. The top row contains data from three sets of metagenome-assembled genomes (MAGs) from different environments. The bottom row displays data from NCBI RefSeq (rarefied to reduce taxonomic bias; see Materials and Methods), RefSeq with only comparisons between genomes annotated as the same species included, and RefSeq with only comparisons between genomes annotated as different species included.

Sets of microbial genomes without the selection biases introduced by isolation were generated from metagenomic studies of three environments: infant fecal samples (1,163 metagenomes collected from 160 hospitalized premature infants over 5 years) (33), the ocean (234 metagenomes collected from the global *Tara* Oceans Expedition over 7 years) (34), and a meadow soil ecosystem (60 metagenomes collected from three depths at five locations for five time points across a grassland meadow) (26). A taxonomically balanced set of genomes from RefSeq was generated by randomly choosing 10 genomes from each of the 480 species in RefSeq with at least 10 genomes (see Table S1 in the supplemental material; see Materials and Methods for details). All genomes within each of the four sets were compared to each other in a pairwise manner using the FastANI algorithm (10). Discrete sequence groups based on both ANI and genome alignment percentages were found in all genome sets (Fig. 1). Notably, species identity gaps were even more prominent in genome sets based on MAGs (metagenome-assembled genomes) than in those from RefSeq (which mainly consists of cultured isolate genomes). Comparisons of RefSeq genomes marked as belonging to the same bacterial species versus different bacterial species showed that the identity gap was largely consistent with annotated NCBI species taxonomy and that most genome clusters segregated from each other with a cluster boundary at around 95%. Thus, the analysis is consistent with prior suggestions that this cutoff delineates the species boundary. MAGs from the human microbiome were often very similar to each other (>98% ANI), whereas MAG clusters from the ocean included greater numbers of divergent strain types. In contrast, most of the comparisons involving genomes from soil involved distinct species.

10.1128/mSystems.00731-19.2 TABLE S1 Information about studied genome sets. Download Table S1, CSV file, 0.01 MB. Copyright © 2020 Olm et al. 2020 Olm et al. This content is distributed under the terms of the Creative Commons Attribution 4.0 International license.

### Gaps in ANI spectra are consistent with measurements of recombination and selection.

We next estimated how the evolutionary forces that could lead to the formation of discrete sequence clusters change with ANI. Estimates for rates of homologous recombination and genome-wide *dN*/*dS* ratios between pairwise genome alignments were calculated in a high-throughput manner (see Materials and Methods for details). Rates of homologous recombination were estimated using the following two methods: (i) analysis of the bias toward genomes sharing longer stretches of identical DNA than expected by random chance (length bias; calculated using previously described methods [35]) and (ii) analysis of the presence of greater numbers of identical genes than would be expected by random chance based on the genome-wide ANI, performed similarly to previously described methods (36). Genome-wide average *dN*/*dS* ratios were calculated for pairs of genomes based on a python implementation of the Nei equation (37).

**FIG 2:**
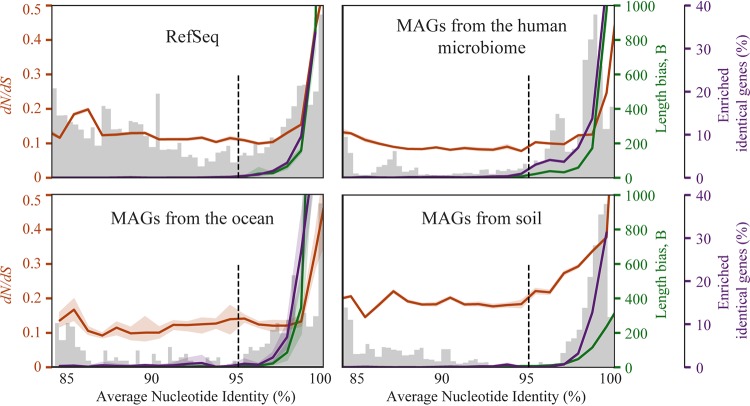
Metrics of recombination and selection follow patterns related to the proposed 95% ANI species threshold. Each plot displays a histogram of ANI values resulting from pairwise comparison within a genome set (light gray bars), the median *dN*/*dS* ratio at each ANI level (orange line), and the median estimated recombination rate at each ANI level determined using two criteria, namely, the percentage of enriched identical genes (purple line; see Materials and Methods for details) and length bias (green line), as measured using the program PopCOGent. A dotted line is drawn at 95% ANI to mark the commonly proposed threshold for species delineation, and 95% confidence intervals are shown shaded around orange, green, and purple lines. Color coding corresponds to *y*-axis labels.

Determinations of both estimated homologous recombination rates and *dN*/*dS* ratios followed consistent patterns in relation to the 95% ANI species threshold in all three measured genome sets (Fig. 2). Estimated homologous recombination rates as measured using both methods showed a sharp decline from 100% ANI to around 95% ANI. This result could have been due to decreases in efficiency of homologous recombination with decreasing sequence similarity.

All genome-wide *dN*/*dS* ratios were below 1, as previously observed for whole-genome *dN*/*dS* comparisons (38). Ratios were highest (∼0.4) between organisms with high sequence similarity and decreased with decreasing ANI, reaching a bottom asymptote of about 0.1 (Fig. 2). Interestingly, the *dN*/*dS* asymptote did not tend to occur at 95% ANI, as in the case of homologous recombination, but earlier, at around 98% ANI. It is well documented that whole-genome *dN*/*dS* values tend to be higher in recently diverged genomes (i.e., those with high ANI values) (24, 38), and it is hypothesized that this is because it takes time for purifying selection to purge nonsynonymous mutations that are slightly deleterious (nearly neutral). MAG clusters from soil showed a slower decline in *dN*/*dS* values with increasing divergence than was observed in other environments.

### Evaluating marker gene thresholds for bacterial species delineation.

**FIG 3:**
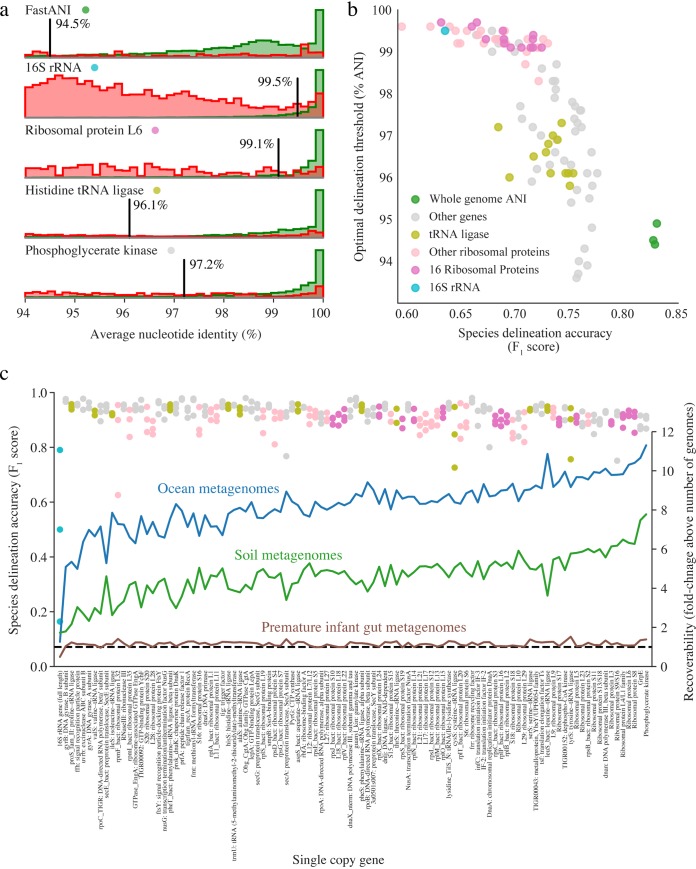
Whole-genome alignment outperforms analysis of marker genes for species discrimination. (a) Histograms of ANI values from comparisons between bacteria from RefSeq annotated as belonging to the same species (green) or different species (red). Each row represents a different method of nucleotide sequence alignment, and vertical black lines indicate the ANI value with the highest F1 score for the corresponding method. (b) Comparison of optimal species discrimination threshold to F1 score for reconstruction of species-level clusters from RefSeq. Whole-genome comparison algorithms, a 16S rRNA alignment, and single-copy gene alignments were tested. (c) Accuracy of marker genes for reconstruction of species clusters based on 95% ANI whole-genome alignments of genomes from metagenomes (dots; left *y* axis) and recoverability of maker genes from metagenomic data from different environments (lines; right *y* axis). A horizontal dotted line marks a recoverability level of 1, meaning equal numbers of marker genes and genomes were assembled from the environment.

To generate an overview of the species composition of an environment, it is necessary to be able to distinguish species from each other. We investigated thresholds for species delineation based on genomes deposited in RefSeq for genome-wide ANI and over 100 marker genes previously identified to occur in single copy in all bacterial genomes (39) (Fig. 3; see also Table S3). These thresholds establish the nucleotide identity shared by genotypes of bacteria considered to be the same species by RefSeq. Genotypes should belong to the same species at values above this threshold and to different species below it. We assigned a score for accuracy of the distinction (F_1_ score) using many methods and found that ANI analysis performed better than analyses based on any single-copy gene (Fig. 3b). Given that whole-genome alignments are generally not possible for all community members, we also ranked genes for their species discrimination ability. The threshold for the 16S rRNA gene was 99%, identical to that recently reported by Edgar and Valencia (40) and significantly higher than the commonly used 97% operational taxonomic unit (OTU) clustering threshold. The discrimination accuracy for the 16S rRNA gene was among the lowest of those determined for the genes considered (Fig. 3b). Among the genes encoding ribosomal proteins, the gene for ribosomal protein L6 had a high F_1_ score and a threshold of <99%, whereas thresholds for tRNA ligase genes and other single-copy genes were generally around 97% to 96% ANI (Fig. 3a and b; see also Table S2).

10.1128/mSystems.00731-19.3 TABLE S2 Ideal thresholds for species-level delineation using RefSeq. Download Table S2, CSV file, 0.01 MB. Copyright © 2020 Olm et al. 2020 Olm et al. This content is distributed under the terms of the Creative Commons Attribution 4.0 International license.

10.1128/mSystems.00731-19.4 TABLE S3 Recoverability of single-copy genes in studied genome sets. Download Table S3, CSV file, 0.02 MB. Copyright © 2020 Olm et al. 2020 Olm et al. This content is distributed under the terms of the Creative Commons Attribution 4.0 International license.

As RefSeq species classifications have errors and taxonomic anomalies (10), we next compared the abilities of marker genes to distinguish MAGs that share >95% whole-genome ANI (Fig. 3c). The accuracy score for most marker genes was high in all three tested MAG genome sets, with the exception of that for the 16S rRNA gene, which performed poorly (Fig. 3c). This was likely due to the gene being frequently misbinned (due to aberrant coverage values resulting from its presence in multiple copies) and/or misassembled (due to fragmentation caused by its highly conserved regions) in metagenomic data.

It is important that genes used to generate species inventories are easily reconstructed from metagenomes; otherwise, species inventories would be incomplete. Thus, we compared the number of marker genes that could be assembled from each data set to the number of genomes that were assembled and binned from the same data set and found that, on average, five times more ribosomal genes than genomes were recovered (Fig. 3c; see also Table S3). This was especially apparent for metagenomes from the ocean and soil, which are complex environments. 16S rRNA genes were recovered much less often than other single-copy genes, as has been previously described, but overall, there was a wide range in the recoverability of marker genes. Finally, we established that over 50% of tested marker genes are present in over 80% of archaeal genomes in RefSeq (Table S5). Thus, while whole-genome comparison methods are the most accurate for species-level characterization, many marker genes are good options for species-level marker gene analysis in studies when genomes were not comprehensively recovered. A table listing recommended ANI thresholds based on 95% whole-genome ANI for the 10 single-copy genes with the highest recoverability is provided (Table 1), and thresholds for all genes are available in the supplemental material (Table S4). An open source-program enabling species-level marker gene analysis from metagenomic assemblies is available on GitHub (https://github.com/alexcritschristoph/RPxSuite).

**TABLE 1:** Species ANI thresholds for the 10 single-copy genes with highest recoverability scores

HMM name	Species ANI threshold (%)	Species delineation accuracy (F1 score)	Recoverability (fold change over no. of recovered genomes)	Bacterial genomes with gene (%)	Bacterial genomes with multiple copies (%)	Present in archaea^*a*^	Description
PGK	95.8	0.9	5.12	95.2	4.8	X	Phosphoglycerate kinase
GrpE	95	0.89	4.87	96	6.8		GrpE
Ribosomal_S8	98.3	0.9	4.44	91.9	1.6	X	Ribosomal protein S8
Ribosomal_L6	97.5	0.92	4.42	91.9	1.6	X	Ribosomal protein L6
Ribosomal_L4	98	0.9	4.35	91.2	1.6	X	Ribosomal protein L4/L1 family
Ribosomal_S9	97.2	0.87	4.29	94.2	1.9	X	Ribosomal protein S9/S16
Ribosomal_L3	98	0.89	4.26	91.2	1.5	X	Ribosomal protein L3
TIGR00663	95.8	0.93	4.25	90.8	3.3		*dnaN* (DNA polymerase III, beta subunit)
Ribosomal_S13	97.9	0.89	4.24	92.8	3.2	X	Ribosomal protein S13/S18
Ribosomal_S11	97.7	0.89	4.22	92.2	1.9	X	Ribosomal protein S11
16S	96.7	0.48	1.38	30.5	56.3	X	16S rRNA gene (full length)

a X, gene present in genome.

10.1128/mSystems.00731-19.5 TABLE S4 Thresholds for species delineation based on 95% whole-genome ANI clustering. Download Table S4, CSV file, 0.01 MB. Copyright © 2020 Olm et al. 2020 Olm et al. This content is distributed under the terms of the Creative Commons Attribution 4.0 International license.

10.1128/mSystems.00731-19.6 TABLE S5 Recoverability of single-copy genes in archaea. Download Table S5, CSV file, 0.01 MB. Copyright © 2020 Olm et al. 2020 Olm et al. This content is distributed under the terms of the Creative Commons Attribution 4.0 International license.

## DISCUSSION

In line with previous studies using reference databases (10) and metagenomic DNA fragments (6), we show here that bacterial diversity in natural communities is clustered in all three environments studied (Fig. 1). Clustering was observed based on both average nucleotide identity and genome alignment fraction (a proxy for shared gene content), estimated rates of horizontal gene transfer fell to near zero at the 95% ANI boundary in all tested environments, and genome-wide *dN*/*dS* ratios consistently leveled near values of 0.15 at around 98% ANI in most environments (Fig. 2). Together, these independent metrics support the existence of naturally distinct “bacterial species.”

The observed drop in estimated homologous recombination with decreasing DNA similarity suggests that sequence-dependent homologous recombination is likely a homogenizing force preventing dissolution of bacterial species, in line with previous experimental laboratory studies, computer simulations (16, 17, 20), and direct measurements of recombination versus mutation rates in natural populations (6, 21). These observations support the notion that bacteria of the same species recombine often due to shared sequence similarity and the notion that rates approach zero as sequence identity decays, leading to species divergence and speciation. However, while both ANI values and the percentages of identical genes shared between genomes approach 0 at around 95% ANI, because it takes time for nucleotide sequences to diverge, recombination may cease at some point above this ANI value.

Given that an increasing number of genomes derive from metagenomic DNA, which does not require culturing or isolation to obtain, a sequence-based method for species delineation is a practical necessity. While thresholds are always prone to exceptions, a genome-wide 95% ANI threshold for species delineation appears to be optimal given the data presented here and previously (8–10) as well as current species-level taxonomic assignments in NCBI. Here, we identified many single-copy genes that can act as effective proxies for whole-genome ANI values and that are well reconstructed from metagenomes using current technologies (Fig. 3; see also Table 1) and thus are useful for descriptions of microbial communities that are resistant to comprehensive genome recovery. While no tested gene was top ranked in all evaluated metrics, ribosomal proteins S8, L6, and L4 are especially promising candidates given their high level of recoverability, average species delineation accuracy, presence in archaea, and history of use in deep phylogenetic trees (41).

## MATERIALS AND METHODS

### Preparation of genome sets.

The following four criteria were used to identify sets of genomes with minimal isolation and selection biases. (i) Genomes must be assembled from DNA extracted directly from the environment without enrichment or culturing. (ii) There must be no preference for particular taxa during metagenomic genome binning and/or curation. (iii) Genomes must be available from at least 50 samples from the same or similar environments, and there must be at least 1000 genomes in total. (iv) All genomes, i.e., not just the dereplicated genome set, must be publicly available for download. Many potential metagenomic studies were disqualified based on criteria iii and iv, leading to the ultimate selection of three genome sets for follow-up analysis (26, 33, 34). Recent studies involving large-scale genome binning (42, 43) could not be included because their predereplication sets included replicate genomes from the same time series, leading to the presence of artificial genome clusters.

In this study, the first analysis set contained 2,178 bacterial genomes from 1,163 premature infant fecal samples, all of which were collected from infants born in the same neonatal intensive care unit (33) (see Table S1 in the supplemental material). These samples are of low diversity, and *Proteobacteria* and *Firmicutes* species accounted for >80% of the bacteria (and, for most samples, >90% of the reads could be assigned to genomes). The second set contained 1,166 genomes from the ocean, including *Bacteria* and *Archaea* (34). The third set contained 1,859 genomes from a meadow soil ecosystem (26) that spanned a large number of diverse phylogenetic groups. We also included 4,800 genomes from NCBI RefSeq, accessed February 2018, where we randomly selected 10 genomes from each of the 480 bacterial species with at least 10 genomes.

All publicly available genomes available in RefSeq as of 21 February 2018 were downloaded using ncbi-genome-download (https://github.com/kblin/ncbi-genome-download) and the “ncbi-genome-download –format GenBank -p 4 bacteria” command. Taxonomy of all genomes was determined using ETE3 (44) based on the provided taxonomy identifier (ID). A genome set consisting of a subset of the entire RefSeq set was generated to balance taxonomic representation—10 genomes were randomly chosen from the 480 species in RefSeq that contained at least 10 species, leading to a total of 4,800 genomes. CheckM (45) was run on all genome sets, and only those with completeness greater than or equal to 70% and less than 5% contamination were retained. All four genome sets are available at https://doi.org/10.6084/m9.figshare.c.4508162.v1.

### Visualization of average nucleotide identity gap.

All genomes in each genome set were compared to each other in a pairwise manner using FastANI (10), and the genome alignment fraction was calculated by dividing the count of bidirectional fragment mappings by the number of total query fragments. ANI values and genome alignment fraction values were averaged for reciprocal comparisons, and comparisons of genomes to themselves were removed. The density of each combination of ANI and alignment fraction was calculated using scipy.stats.kde (46). The density was plotted in a 3-dimensional histogram using matplotlib (47).

### Calculation of *dN*/*dS*.

dRep (14) was used for comparisons of all genome sets in a pairwise manner on a gene-by-gene basis using the “dRep dereplicate –S_algorithm goANI -pa 0.8 -con 5 -comp 70” command. Briefly, this identifies open reading frames using Prodigal (48) and compares their nucleic acid sequences using NSimScan (49). The script “dnds_from_drep.py” was used to calculate the dN/dS ratio among aligned sequences (https://github.com/MrOlm/bacterialEvolutionMetrics). This involves first aligning the amino acid sequences encoded by pairs of genes with which at least 70% of the genes aligned with at least 70% sequence identity and which were reciprocal best hits. Sequences were aligned globally using the BioPython Align.PairwiseAligner (50) with a blosum62 substitution matrix, a −12 open gap score, and a −3 extend gap score. The alignment was then converted into a codon alignment using biopython, and the numbers of synonymous sites, synonymous substitutions, nonsynonymous sites, and nonsynonymous substitutions were recorded. Finally, the overall dN/dS ratio was calculated for each genome alignment using the following formula:

dN/dS=(nonsynonymous substitutions/nonsynonymous sites)/(synonymous substitutions/synonymous sites)

### Calculation of estimated homologous recombination.

Rates of homologous recombination between genome pairs were calculated using two methods: (i) calculation of the bias toward longer stretches of identical DNA than expected by random chance and (ii) calculation of the bias toward identical genes based on the overall ANI between genome pairs (determined in a manner similar to previously described methods [36]).

The length bias toward longer stretches of identical DNA sequences between genome pairs was measured using the program PopCOGent (reported as “Observed SSD”) (35).

To calculate the bias toward identical genes, or the percentage of “enriched identical genes,” the set of genes aligned between the genomes in each pair was first filtered to include only those with at least 500 bp aligned. Genome pairs in which fewer than 1,000 genes were aligned were excluded from this analysis. The probability of gene alignments being identical by chance was determined using the following formula:

$$\text{overall genome}\;{\text{ANI}}^{\left(\text{length of gene alignment}\right)}$$

The expected number of identical genes for a genome pair was calculated as the probability of the individual gene alignments being the same (using the formula presented above) multiplied by the number of aligned genes. The determined number of identical genes for each genome pair was calculated as the number of alignments with 100% average nucleotide identity. Finally, the genome-wide bias toward identical genes was calculated using the following formula:

hr=(a–e)/i

where hr is the estimated degree of homologous recombination (“enriched identical genes”), *a* is the actual number of identical genes, *e* is the expected number of identical genes, and *i* is the number of aligned genes with at least 500 bp aligned. A plot comparing enriched, expected, and actual percentages of identical genes is provided in Fig. S1 in the supplemental material. Source code is available as python notebooks at https://github.com/MrOlm/bacterialEvolutionMetrics.

10.1128/mSystems.00731-19.1 FIG S1 Actual, expected, and enriched identical genes in relation to average nucleotide identity. Each plot displays a histogram of ANI values resulting from pairwise comparison within a genome set (light gray bars), the median percentage of enriched identical genes at each ANI level (purple line), the median actual percentage of identical genes at each ANI level (green line), and the expected percentage of identical genes at each ANI level (red line). A dotted line is drawn at 95% ANI to mark the commonly proposed threshold for species delineation, and 95% confidence intervals are shown with shading around orange, green, and purple lines. Download FIG S1, PDF file, 0.2 MB. Copyright © 2020 Olm et al. 2020 Olm et al. This content is distributed under the terms of the Creative Commons Attribution 4.0 International license.

### Marker gene identification and clustering.

Bacterial single-copy genes were identified based on a previously curated set of hidden Markov models (HMMs) for 107 genes expected to be at single copy in all bacterial cells (39), as accessed on GitHub on 10 April 2019 at https://github.com/MadsAlbertsen/multi-metagenome/blob/master/R.data.generation/essential.hmm. The amino acid sequences of all genomes in all four genome sets were annotated using prodigal in “single” mode (48) and searched against the single-copy-gene HMMs using the command “hmmsearch -E 0.001 –domE 0.001” (hmmer.org). All hits with scores above the trusted cutoff value for each HMM were retained. Nucleic acid sequences for each hit were compared using usearch (51) and the “usearch -calc_distmx” command. Genes which were identified in less than 85% of genomes in our RefSeq data set were excluded from further analysis.

16S rRNA genes were identified using SEARCH_16S (52) and the specific “usearch -search_16s -bitvec gg97.bitvec” command. gg97.bitvec was created using the commands “usearch -makeudb_usearch 97_otus.fasta -wordlength 13” and “usearch -udb2bitvec” based on the Greengenes reference database (as accessed at https://github.com/biocore/qiime-default-reference/blob/master/qiime_default_reference/gg_13_8_otus/rep_set/97_otus.fasta.gz) (53). Identified 16S rRNA genes were aligned to each other using Mothur (54) with RDP (release 11, update 5 [55]) used as the template. Distance matrices for 16S genes were calculated using the Mothur dist.seqs command.

Recoverability was calculated based on the total number of gene copies that could be recovered from a given metagenomic assembly for each marker gene. This is impacted by the assembly algorithm, the sequencing technology, and the nucleotide sequence being assembled. For each set of metagenome-assembled genomes (MAGs), the number of filtered genomes was set at 100% recoverability. The recoverability of each single-copy gene was calculated for each set of MAGs using the following equation:

recoverability=(number of assembled genes)/(number of filtered genomes)

For example, if 100 genomes and 300 sequences of gene *x* were recovered from a set of metagenomes, the recoverability of gene *x* would be 3.

The presence of bacterial single-copy genes in archaea was determined using all archaeal genomes present in RefSeq as of 5 March 2018, as accessed using the “ncbi-genome-download –format GenBank -p 4 archaea” command. Single-copy genes were identified using the HMM-based methodology described above, and genes present in at least 80% of archaeal genomes were marked as being “present” in archaea.

### Species delineation thresholds.

Optimal thresholds for species delineation were empirically determined based on pairwise genome distance matrices. For each genome comparison method, all distance thresholds between 80% and 100% were tested, incrementing by 0.1% (80%, 80.1%, 80.2%, etc.). Each pair of genomes at least as similar as the threshold were considered to belong to the same species, and the remaining pairs were considered to belong to different species. A species delineation accuracy value was calculated for each threshold using the F_1_ score, and the threshold with the highest score was considered optimal.

Species delineation accuracies were calculated based on the ability to recreate species-level clusters, first as defined by RefSeq taxonomy annotations. A pairwise matrix was established listing each pair of genomes in our RefSeq genome set and whether or not the pair belonged to the same taxonomic species. Recall, precision, and F_1_scores were calculated for each genome set clustering as follows:

recall=(number of genome pairs correctly identified as belonging to the same species)/(true number of genome pairs belonging to the same species)

precision=(number of genome pairs correctly identified as belonging to the same species)/(number of genome pairs correctly or incorrectly identified as belonging to the same species)

F1 score=2 * [(recall * precision)/(recall+precision)]

Species delineation accuracies were also calculated based on the ability to recreate genome clusters as defined by 95% genome-wide ANI similarity (calculated using FastANI). F_1_ scores were calculated for all thresholds as described above, and the optimal threshold was defined as the threshold with the highest average F_1_ score among the three MAG genome sets. Implementation details are available in python notebooks at https://github.com/MrOlm/bacterialEvolutionMetrics.

### Data availability.

Details of *dN*/*dS* and homologous recombination analyses are available at https://github.com/MrOlm/bacterialEvolutionMetrics, an open-source program enabling marker gene analysis from metagenomic data is available at https://github.com/alexcritschristoph/RPxSuite, and nucleotide sequences of genome sets used in this study are available at https://doi.org/10.6084/m9.figshare.c.4508162.v1.
