# Supplementary figures and images for: Consistent Metagenome-Derived Metrics Verify and Delineate Bacterial Species Boundaries
Source: mSystems. 2020 Jan 14;5(1):e00731-19. doi: 10.1128/mSystems.00731-19 (PMC6967389; doi:10.1128/mSystems.00731-19)

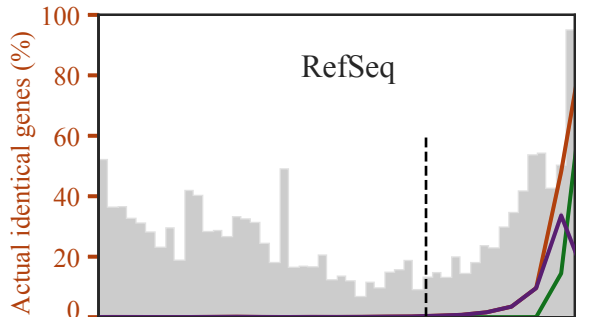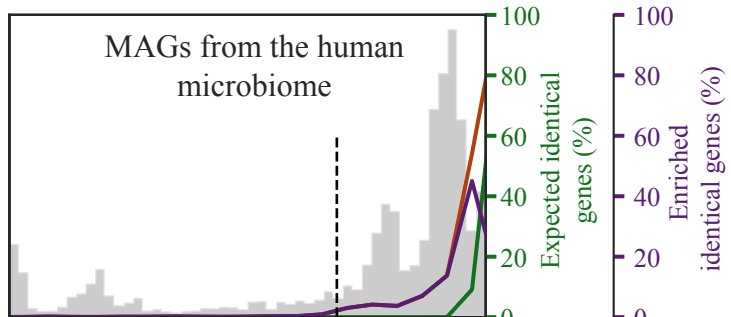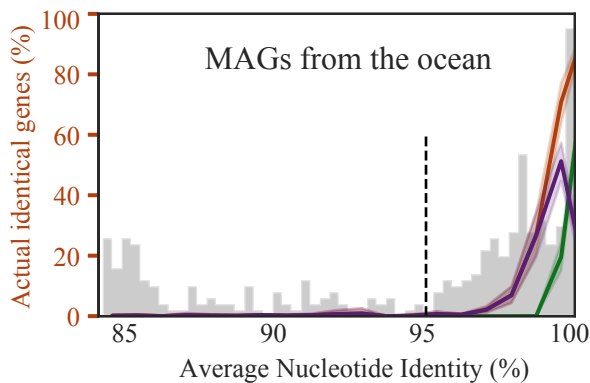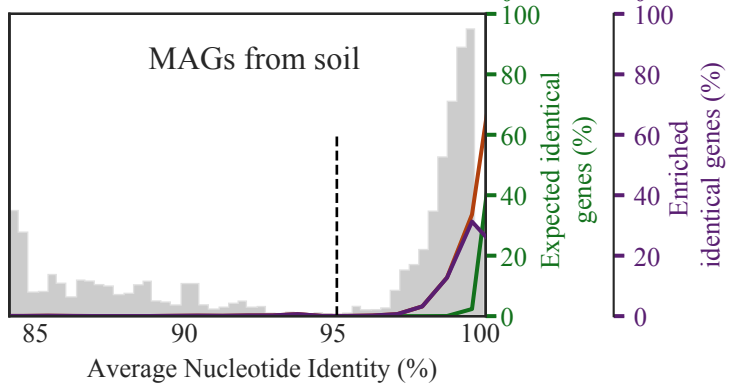

Supplement: FIG S1 [file mSystems.00731-19-sf001.pdf]
